# Enhanced electrochemical and optical properties of polyaniline-based Ag_2_O/NiO/ reduced carbon black nanocomposites for renewable energy applications

**DOI:** 10.1038/s41598-026-69315-7

**Published:** 2026-09-15

**Authors:** Rasha A. Baseer, Alia H. Salama, Amal M. Abdel-karim, Abas A. Yehia, Noha Elhalawany

**Affiliations:** 1 Polymers and Pigments Department, National Research Centre, Giza, 12622 Egypt; 2 Physical Chemistry Department, National Research Centre, Giza, 12622 Egypt

**Keywords:** Polyaniline, Carbon black, Ag/Ni/roCB@PANi, Cyclic voltammetry, Electrochemical behavior, Chemistry, Energy science and technology, Materials science, Nanoscience and technology

## Abstract

A multi-step synthesis strategy was employed to fabricate Ag_2_O/NiO/rOCB@PANI core-shell nanocomposites. Silver oxide and nickel oxide nanoparticles were first decorated onto a reduced oxidized carbon black matrix (Ag/Ni/rOCB hybrid core), followed by the in situ polymeric encapsulation of this core within a protective, conductive polyaniline (PANI) shell, assessing their electrochemical, dielectric, and optical properties for potential applications in renewable energy applications. Structural and spectroscopic analyses, including FTIR, Raman spectroscopy, XRD, SEM/EDAX, and TEM, confirm the successful formation of conductive Ag_2_O/NiO/roCB@ PANi nanocomposites. The electrochemical behavior of the Ag_2_O/NiO/roCB@ PANi composite was investigated using cyclic voltammetry (CV) measurements which identified distinct oxidation and reduction peaks, offering valuable insights into redox behavior. Electrochemical impedance spectra EIS has shown a low charge transfer resistance (Rct), indicating efficient electron transfer at the electrode/electrolyte interface, which are suitable for hydrogen evolution reaction HER. The combined CV and EIS results establish a stronger correlation between the structure of the composite and its electrochemical behavior. The observed enhancement is attributed to the combined contributions of the electroactive PANI matrix, Ag_2_O/NiO components, and the highly conductive roCB, which together promote efficient charge transport and interfacial redox activity. The dielectric study highlights a strong frequency-dependent variation in the dielectric constant (ε′), demonstrating enhanced charge storage capacity and interfacial polarization due to Ag_2_O/NiO/roCB doping. Ac conductivity measurements of the prepared nanocomposites showed high ac conductivity reaching up to 5.21*1 0^− 1^ S/cm^− 1^. AC conductivity (σ_ac_) follows the correlated barrier hopping (CBH) model, indicating improved charge transport and conductivity. Moreover, fluorescence microscopy (FM) has been used to track the optical characteristics of the produced nanocomposites after they are exposed to various lights of different wavelengths. The FM results revealed that the nanocomposites emit fluorescent lights upon irradiation by red, green and blue lights. This suggests that the produced nanocomposites contain fluorophores that are both light-absorbing and capable of longer-wavelength light reemission. These characteristics support their application in LEDs, solar cells, and electronic devices. The findings suggest that Ag_2_O/NiO/roCB@ PANi composites possess promising electrochemical and optical properties, making them viable materials for hydrogen evolution reaction (HER) and renewable energy technologies. As far as we know, no one in the field of electrochemistry has ever created such composites in terms of their preparation method and unique electro catalytical and optical properties.

## Introduction

 One environmentally beneficial way to address the energy needs of human activities is through the use of renewable energy sources. These energy sources are greener, less carbon-intensive, and insensitive to large fluctuations in the market. Hydrogen production is an important category of renewable energy resources which is sustainable substitutes to fossil fuels^1^.

As a result, high-performance energy storage materials are needed to satisfy the rising demand for electrical energy^2^, which is commonly used in electronic devices and accessories.

Carbon nanotubes, graphene, and activated carbons are carbonaceous compounds that have shown outstanding efficacy in mitigating environmental contamination and the energy crisis. These three carbonaceous materials are excessive adsorption resources for energy storage because of their large specific surface areas, strong mechanical strength, and superior electrical conductivity^3^.

Carbonaceous materials are crucial for promoting operative and long-lasting electrochemical processes^4–6^ due to their superior electrical conductivity, large specific surface area, extraordinary structural stability, and affordability^1,7^.

Because of their vast surface areas and distinct chemical and physical properties, carbon nanotubes (CNTs), graphene, and activated carbons (ACs) are considered the best options among these materials^8–10^.

A new family of carbon-only compounds called fullerenes, which were initially known as buckminster fullerenes and include 60 carbon atoms (C60) grouped in a soccer-ball configuration, are also carbon-based materials^11^. However, due to their higher price compared to two-dimensional graphene and one-dimensional carbon nanotubes (CNTs), they have received less attention than other carbon materials^12^.

Conducting polymers have emerged as important carbon precursors for synthesizing carbon-based nanomaterials due to their stable conjugated structures, which enable efficient carbonization while preserving structural integrity^13,14^. In hydrogen and oxygen electrocatalysis, these materials offer notable advantages, including enhanced electrical conductivity from graphitic carbon formation, precise molecular-level compositional control, and uniform heteroatom doping (e.g., N from PANi^15^ and PPy^16^, or S from PTh^17^ that introduces additional active sites. Moreover, their tunable morphology and porosity improve active-site exposure and mass transport, while their rich functional group enables the formation of uniform nanocomposites with various catalytic species, leading to strong synergistic effects. Consequently, conducting polymer derived carbon materials hold significant promise for advanced electrocatalytic applications.

Moreover, the synergistic interaction between metal-based electrocatalysts and carbon supports, including carbon nanotubes, graphene, carbon quantum dots, and nanofibers, significantly enhances electrocatalytic activity and stability by establishing strong electrical and chemical linkages^18^. Additionally, the distinct electronic structures of transition metals play a crucial role in electrocatalysis^19,20^. The hydrogen evolution activity of transition metal-based carbon composites is influenced by the core metals (Ni, Co)^21,22^, nanostructural design^22^, and the presence of a carbon shell^23^.

In addition, the role of metal catalysts in HER is well documented, with nickel (Ni) and silver (Ag) being widely investigated for their electrocatalytic properties. Nickel is commonly used due to its affordability and strong catalytic performance in acidic conditions, while silver improves charge mobility and electron transport within electrode composites^24^.

This study focuses on the development of high-performance Ag_2_O/NiO/roCB@ PANi nanocomposites for hydrogen evolution reaction (HER). The synthesis process begins with the oxidation of carbon black (CB) using the Hummers method, followed by UV-assisted reduction to yield activated carbon (roCB). The Ag_2_O/NiO/roCB composite is prepared by incorporating NiCO3.Ni (OH)2.H2O and silver perchlorate, with subsequent sonication and homogenization steps. Finally, PANi polymerization completes the formation of the nanocomposite. The novelty of this research lies in integrating PANI with varying ratios of a low loading of Ag_2_O/NiO metal oxides supported on an inexpensive activated carbon-based material (roCB) to develop a high-performance nanocomposite for HER applications. To the best of our knowledge, no one in the field of electrochemistry has ever produced composites with such special optical, electrocatalytic, and preparation techniques.

## Materials and methods

### Materials

Carbon Black (CB) and H_2_SO_4_ were purchased from (Elnasr Co., Egypt), KMnO_4_ and Silver perchlorate were purchased from (Sigma-Aldrich, London). NiCO_3_.Ni(OH)2.H_2_O was purchased (ROTH, Karisruhe), Aniline was purchased from (Alpha Chemika, India), Potassium persulfate (PPS) was purchased from (SD Fine-Chem Limited, India).

### Methods

#### Synthesis of oxidized carbon black

Oxidized Carbon Black (oCB) was synthesized using the Hummers method^1^. In this process, 5 g of carbon black was mixed with 200 mL of concentrated H_2_SO_4_ and stirred for 20 min. Subsequently, 30 g of KMnO_4_ was gradually added dropwise, followed by stirring for 1 h. The mixture was then sonicated for 30 min and heated at 90 °C for 3 h. Afterward, 40 mL of 30% H_2_O_2_ was introduced, and stirring continued for another 30 min. The resulting product was washed multiple times via centrifugation until the pH was adjusted to 6.5. Finally, the sample was dried at 70 °C for 24 h.

#### Synthesis of reduced oxidized carbon black (roCB)

Reduced carbon black oxide (roCB) was obtained by subjecting a pre-weighed oCB solution to UV irradiation for 24 h.

#### Synthesis of Ag_2_O/NiO/roCB

For the synthesis of Ag_2_O/NiO/roCB, 50 mL of distilled water containing pre-weighed roCB was sonicated for 10 min. Then, 0.06 g of NiCO_3_.Ni(OH)2.H_2_O was added to the roCB solution and homogenized for an additional 10 min. Next, 10 mg of silver perchlorate was introduced, followed by further homogenization for 15 min. The resulting slurry mixture was washed multiple times via centrifugation and subsequently dried at 70 °C for 24 h.

#### Synthesis of Ag_2_O/NiO/roCB@ PANi

To prepare the Ag_2_O/NiO/roCB@ PANi composites, different amounts of Ag_2_O/NiO/roCB (100, 200, 300, 400, and 500 mg) were added to a suspension containing 0.5 M aniline in 1 M HCl and stirred for 20 min to ensure homogeneous dispersion. Subsequently, 0.3 g of PPS was added to the reaction mixture to initiate polymerization, and the mixture was stirred overnight. The resulting precipitate was collected by filtration, thoroughly washed several times with distilled water to remove unreacted species and residual reagents, and then dried at 80 °C for 24 h.

#### Cyclic voltammetry measurements

Cyclic voltammetry (CV) was employed to estimate the oxidation and reduction potentials of the Ag_2_O/NiO/roCB@ PANi composites. Cyclic voltammetry measurements were carried out in a conventional three-electrode cell, with a saturated Ag/AgCl electrode serving as the reference, and platinum sheet was used as working electrode, while a platinum wire as counter electrode. Before each measurement, the platinum working electrode was polished with alumina paste, rinsed with deionized water, and ultrasonically cleaned to ensure a fresh and reproducible electrode surface. The electrochemical test was performed using an Autolab Potentiostat/ Galvanostat 302 N, and the resulying I-V curves were recorded.

For each test, a fixed quantity of the Ag_2_O/NiO/roCB@ PANi composite with different loading amount of Ag_2_O/NiO/roCB (100–500 mg) was dispersed in 0.1 M potassium chloride solution. Cyclic voltammetry was carried out within the selected potential window at scan rates 5 to 500 mVs^− 1^. The measured currents were normalized to the geometric area of the working electrode.

#### Dielectric and ac-electrical conductivity measurements

Dielectric and AC electrical conductivity properties of the prepared compounds were analyzed using pelletized samples measuring approximately 10 mm in diameter, with thicknesses ranging from 1.57 to 3.54 mm. These pellets were formed by pressing powdered samples under a pressure of 10 tons/cm². Electrical measurements were performed using a HIOKI 3532-50 LCR-Hi Tester.

### Chemical structure and morphology characterization

#### Fourier-transform infrared (FTIR)

The chemical structures of fabricated nanomaterials, hydrogels, and gauze fabric were determined by Fourier Transformation Spectroscopy (FTIR) spectra at room temperature using the Attenuated Total Reflection (ATR) unit attached with FTIR-Vertex 70 Bruker, Germany, in the range of 4000 –400 cm^‒1^.

#### Raman spectroscopy

To identify molecules and study molecular structures, the Raman spectroscopy will be applied. Raman Spectra will be examined by Witec alpha 300 R confocal Raman microscope. The samples are excited by a 532 nm laser at room temperature by a 532 nm laser at room temperature.

#### X-ray diffraction

The crystalline and amorphous nature of the prepared composites will be investigated on an X-ray diffractometer using CuK radiation source.

#### Morphology studies (SEM)

The SEM Model Quanta 250 FEG (Field Emission Gun) is equipped with an EDAX unit for Energy Dispersive X-ray Analysis, operating at an accelerating voltage of 30 kV. It offers magnification ranging from 14x to 1,000,000x, with a resolution of 1 nm for the emission gun.

#### TEM

High-resolution Transmission Electron Microscopy (TEM) was performed using the JEOL-JEM 2100 (Tokyo, Japan) to analyze the structural characteristics of the prepared materials.

#### Fluorescence microscopy

The test has been done using fluorescence microscope, with filter cubes A4, I3 and N2.1,

Leica Company, Germany, at Microbiology Research Lab., Cairo University.

## Result and discussion

**Fig. 1:**
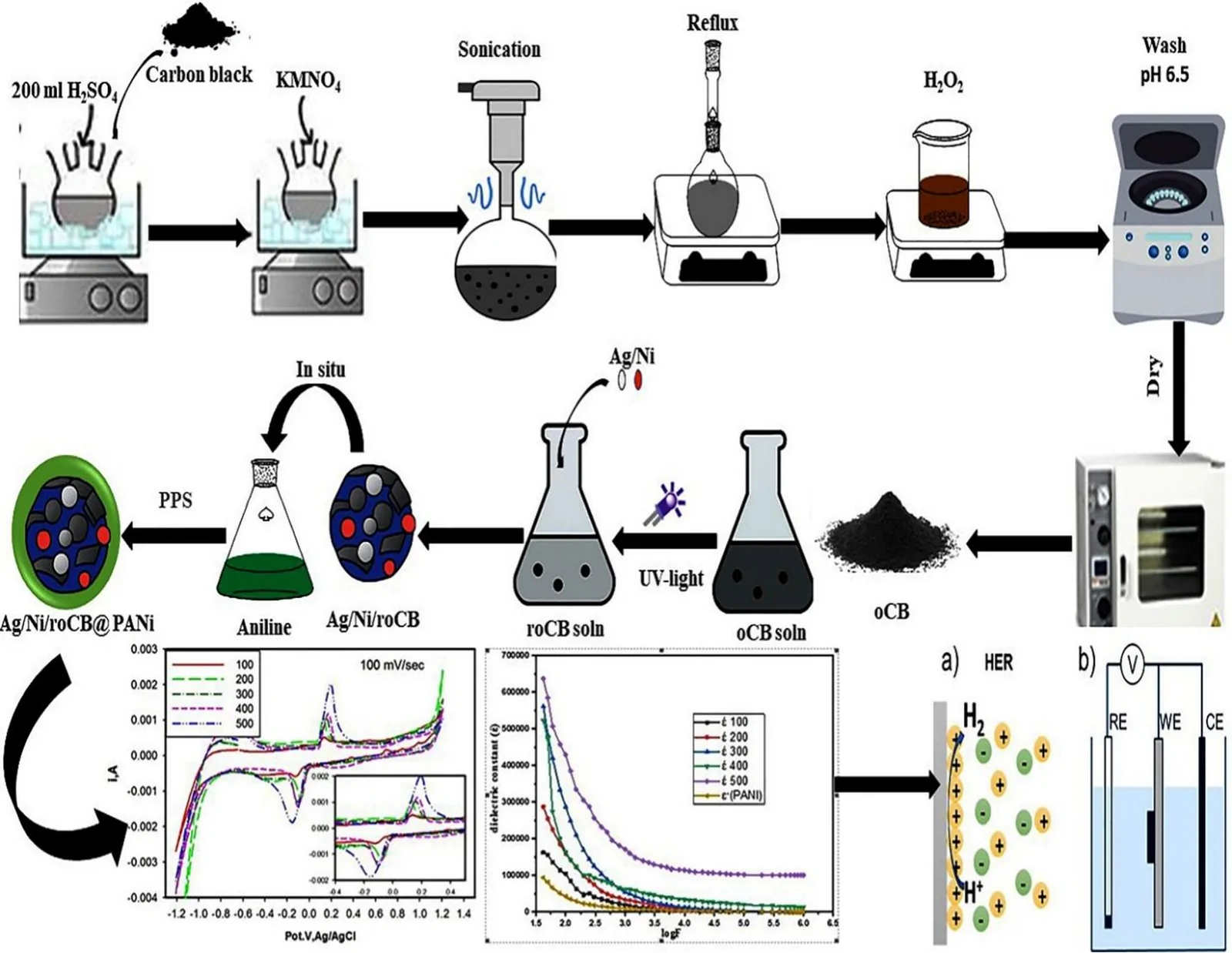
Graphical illustration of the synthesis strategy and HER performance of the Ag_2_O/NiO/roCB@ PANi composite.

**Fig. 2:**
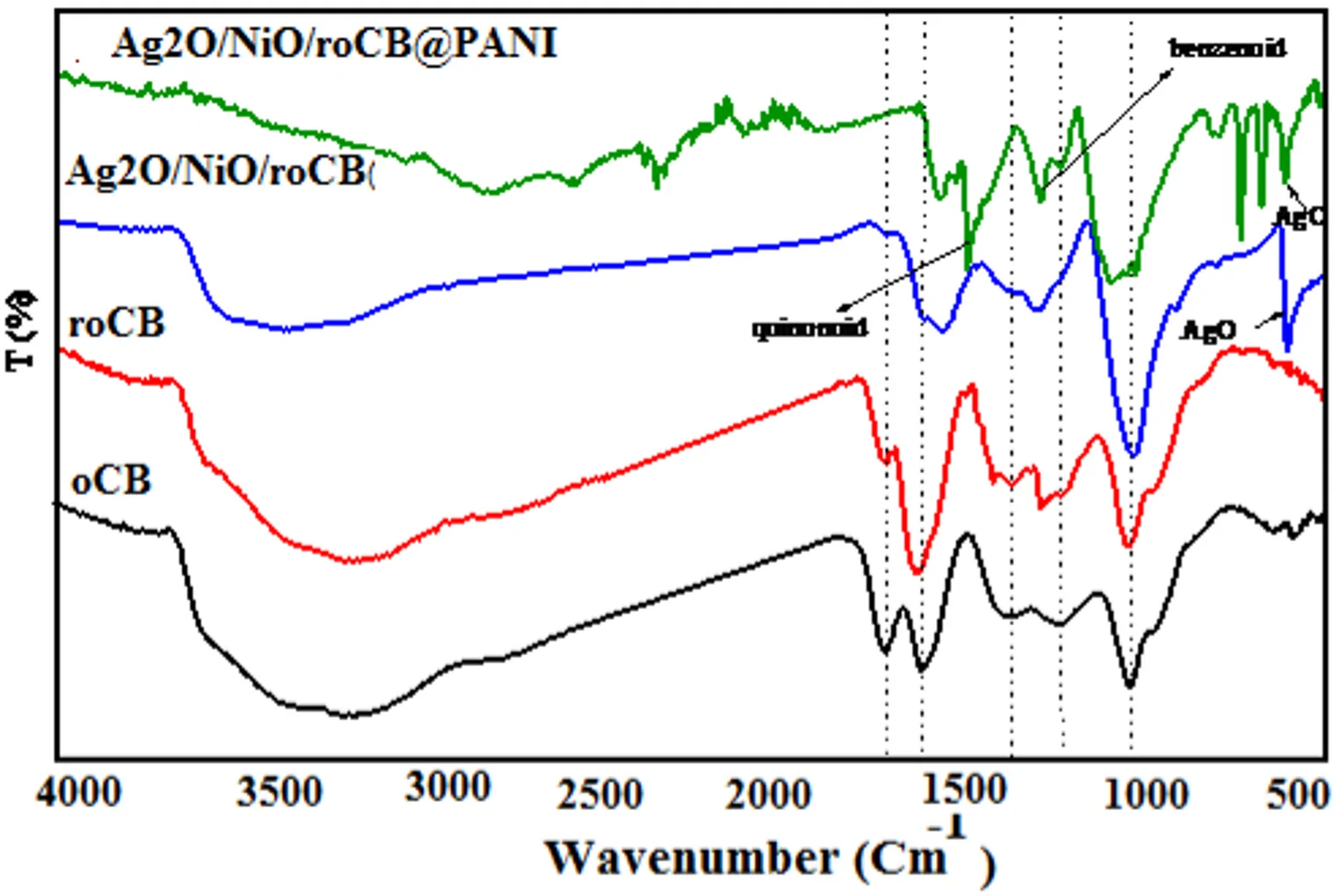
FTIR of oCB, roCB, Ag_2_O/NiO/roCB, and Ag_2_O/NiO/roCB@ PANi.

The HER electrocatalyst composite was synthesized through a multi-step process, beginning with the preparation of reduced oxidized carbon black (roCB), followed by the formation of Ag/Ni/roCB, and culminating in the synthesis of the Ag/Ni/roCB@PANi composite, as illustrated in Fig. 1. **T**he FTIR spectra of oCB, roCB, Ag_2_O/NiO/roCB, and the final Ag_2_O/NiO/roCB@ PANi nanocomposite (Fig. 2) provide definitive molecular-level evidence for successful multi-step synthesis, chemical reduction, and strong interfacial coupling among all constituent materials. The spectrum of oxidized carbon black (oCB) initially exhibits prominent, broad absorption bands at approximately 3486 cm^− 1^ and 1715 cm^− 1^, corresponding to O-H hydroxyl stretching and C = O carboxyl/carbonyl stretching modes, respectively, alongside characteristic C-O-C ether vibrations^25,26^. Upon UV-assisted reduction to roCB, the intensities of these oxygen-bearing functional groups decrease markedly. This sharp attenuation of C = O and O-H bands confirms the efficient removal of oxygen moieties and the successful restoration of the sp^2^ hybridized carbon network necessary for high electronic conductivity.

The spectrum of Ag_2_O/NiO/roCB displays an absorption peak at 615 cm^− 1^, which arises due to the interaction between NiO and AgO^27,28^. Furthermore, a noticeable decrease in the intensity of the carbonyl peak indicates an enhanced reduction process following modification with silver perchlorate and NiCO_3_.3Ni(OH)_2_.2H_2_O.

The FTIR spectrum of Ag_2_O/NiO/roCB@ PANi exhibits characteristic absorption bands at 1565 cm^− 1^ and 1487 cm^− 1^, corresponding to the stretching vibrations of the quinonoid and benzenoid rings^23^. A prominent peak at 809 cm^− 1^ indicates the presence of para-distributed aromatic rings, confirming polymer formation. The peak at 1226 cm^− 1^ is attributed to C–N stretching within the benzenoid ring. Additionally, bands in the 1023–1091 cm^− 1^ region are due to overlapping signals from vinyl ether groups in roCB and in-plane C–H bending vibrations, while the peak at 797 cm^− 1^ originates from out-of-plane C–H bending vibrations^29^. Notably, the formation of the Ag_2_O/NiO/roCB@ PANi nanocomposite is further supported by the observed spectral shifts relative to pristine PANi. Both the quinonoid (C = N) and benzenoid (C = C) characteristic bands shift toward lower wavenumbers, indicating strong interfacial interactions between the conjugated PANi chains and the restored sp²-carbon domains of roCB. This red shift can be attributed to enhanced π–π interactions and increased electronic coupling at the PANi/roCB interface, which promote charge delocalization along the conjugated backbone. These interactions provide spectroscopic evidence for the successful integration of roCB within the PANi matrix and contribute to the modified electronic structure of the resulting nanocomposite.

**Fig. 3:**
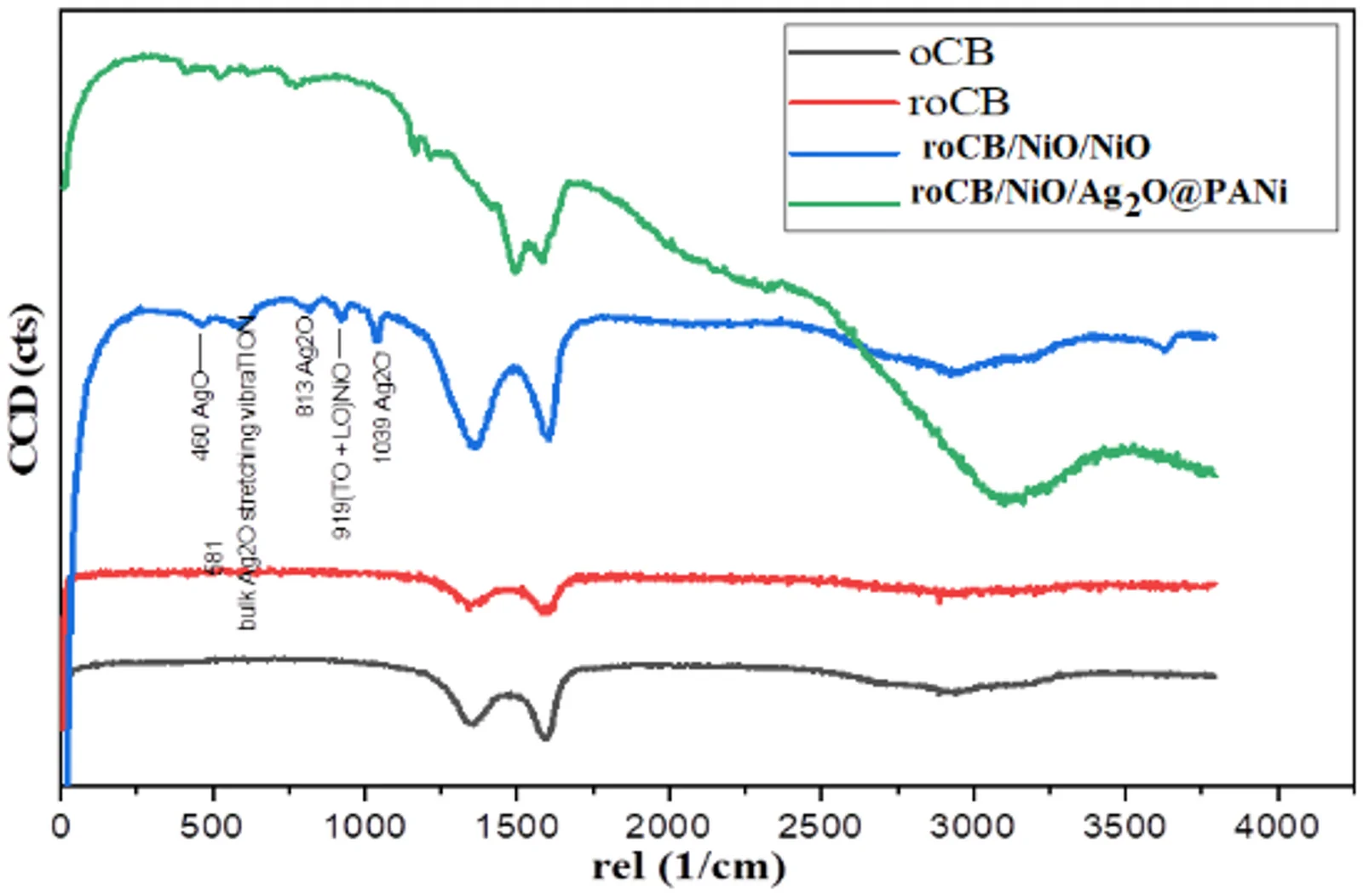
Raman spectroscopy of oCB, roCB, Ag_2_O/NiO/roCB, and Ag_2_O/NiO/roCB@ PANi.

Figure 3 presents Raman spectroscopy data for oCB, roCB, Ag_2_O/NiO/roCB, and Ag_2_O/NiO/roCB@ PANi. Raman spectroscopy serves as a powerful tool for analyzing carbon-based materials. The spectra of both oCB and roCB exhibit two dominant peaks: the G (graphite) peak, observed between 1601 and 1592 cm^− 1^, signifies sp^2^ hybridized carbon, whereas the D (disorder) peak, located around 1351–1336 cm^− 1^, represents sp^3^ hybridized carbon^30^. The ID/IG ratio of oCB and roCB was measured at 0.93 and 0.98, respectively, indicating a decline in the order of the graphitic structure following the reduction of oCB.

The Ag/Ni/roCB spectrum exhibits pronounced G and D bands at 1601 cm^− 1^ and 1359 cm^− 1^, respectively. The ID/IG intensity ratio increased from 0.98 in roCB to 1.04 in the Ag/Ni/roCB composite. This notable increase in the ID/IG ratio primarily reflects an elevated degree of structural disorder and defect site generation within the sp^2^ carbon network, which arises from the physical disruption of graphitic domains and strong interfacial interactions during the loading of Ag and Ni species. While chemical reduction of carbon black is directly verified by the attenuation of oxygen-bearing functional groups in FTIR spectra which directly tracks the chemical modifications during synthesis; the severe attenuation of bands associated with oxygenated species C = O at 1715 cm^− 1^ and C–O at 1050 cm^− 1^ confirms effective chemical reduction, while new low-frequency vibrations 600 cm^− 1^ signal metal-ligand/oxide interaction, the elevated ID/IG ratio confirms that the subsequent metallic incorporation introduces additional surface defects and structural heterogeneity, which are beneficial for exposing active sites for the hydrogen evolution reaction (HER). This suggests further structural disorder within the graphitic framework after modification with silver perchlorate and NiCO_3_.Ni(OH)_2_.H_2_O, confirming additional reduction of roCB as observed in FTIR analysis. A broad G’ peak at 2924 cm^− 1^ appears as an overtone, influenced by contributions from D + G phonons^30^. Other key peaks are noted at 463, 581, 813, 919, and 1042 cm^− 1^, corresponding to AgO, bulk Ag_2_O stretching vibrations, Ag_2_O, NiO (TO + LO), Ag_2_O, respectively^18,31^. In addition to, weak band centered at 3631 cm^− 1^ corresponding to (α-Ni(OH)_2_, O-H stretch).

Raman spectra of Ag_2_O/NiO/roCB@ PANi reveal distinct shifts in the G and D bands due to their interaction with PANi absorption bands. The G and D peaks, appearing at 1587 cm^− 1^ and 1492 cm^− 1^, correspond to δN-H (semi-quinonoid) and υC = N (imine) vibrations of PANi^13^. A broad G’ band is observed at 3099 cm^− 1^. Additional significant peaks at 524, 631, 771, 1162, and 1216 cm^− 1^ are attributed to γN-H, δRing (benzenoid ring), δRing (quinonoid ring), δC-H (semi-quinonoid), and υC-N (amine) vibrations, respectively^13^.

**Fig. 4:**
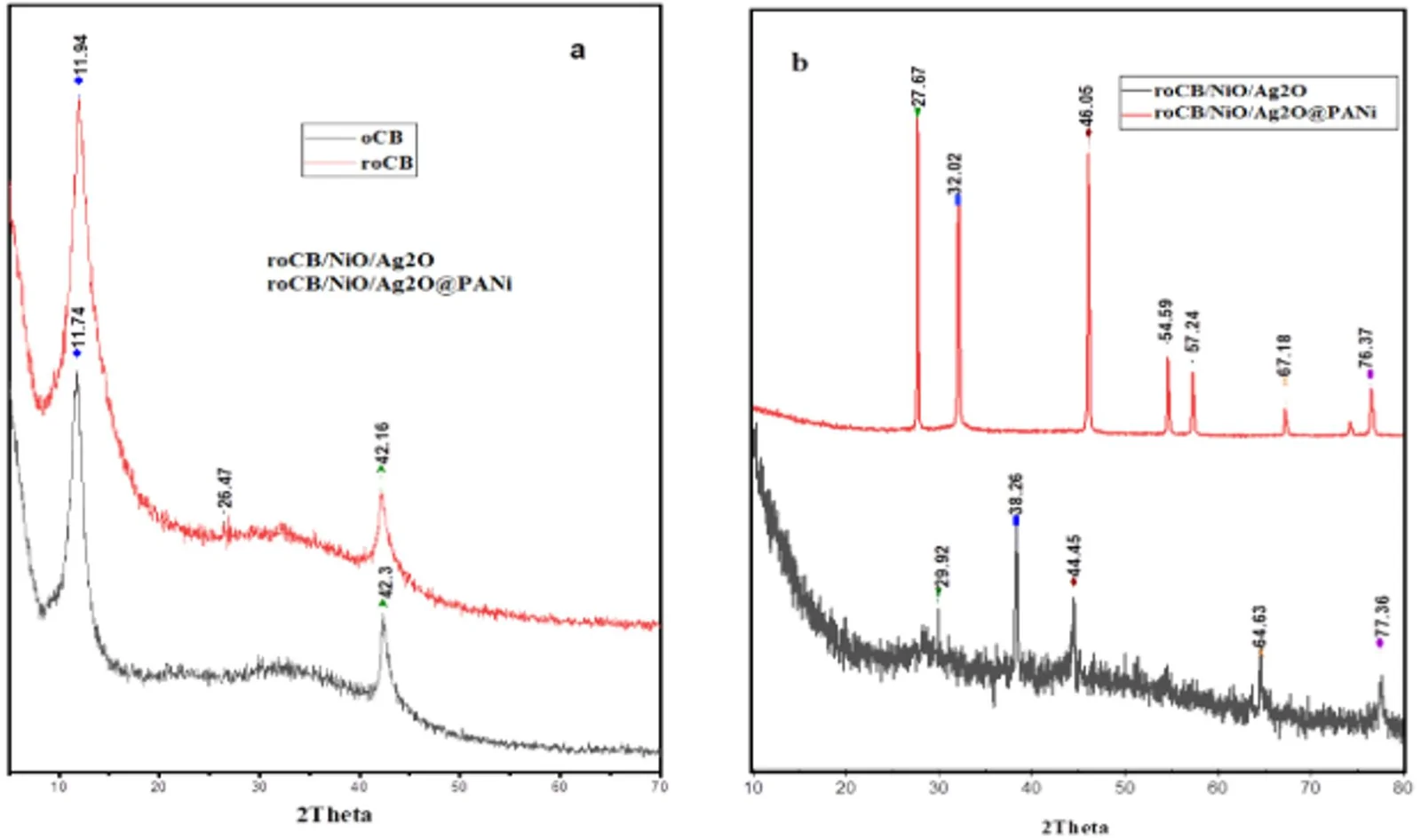
XRD spectra of oCB, roCB, Ag_2_O/NiO/roCB, Ag_2_O/NiO/roCB@ PANi and the spectra of oCB and roCB.

Figure 4 illustrates the XRD spectra for oCB, roCB, Ag_2_O/NiO/roCB, and Ag_2_O/NiO/roCB@ PANi. The spectra of oCB and roCB display a sharp peak at approximately 11.74^°^ and 11.94^°^, respectively, corresponding to the (001) basal plane of graphene structures^14^. The presence of this peak suggests effective exfoliation of the graphitic structure and interaction of oxygen-containing functional groups between graphitic layers^15^. Additionally, a peak at approximately 42^°^ is attributed to the (100)/(101) CB graphitic fraction^16^. Upon reduction, a small hump appears at 26.47^°^ in the roCB spectrum, associated with the (002) CB graphitic plane^16,17^. The XRD spectrum of Ag_2_O/NiO/roCB reveals a (002) reflection around 29°, characteristic of roCB. Furthermore, peaks corresponding to Ag_2_O are observed at 2θ = 38.31^°^, 44.4^°^, 64.58^°^, and 77.36^°^, associated with the (111), (200), (202), and (311) planes of cubic silver. These peaks interfere with those of NiAg_0.40_ 3D porous nanoclusters, which exhibit reflections at 44.4^°^ and 77.36^°^ due to the (111) and (220) planes of cubic nickel^19^.

The main 2θ bands of Ag_2_O/NiO/roCB shift to 27.67^°^, 32.02^°^, 46.05^°^, 67.18^°^, and 76.37^°^ in Ag/Ni/roCB@PANi, with additional new peaks appearing at 54.59^°^ and 57.24^°^.

**Fig. 5:**
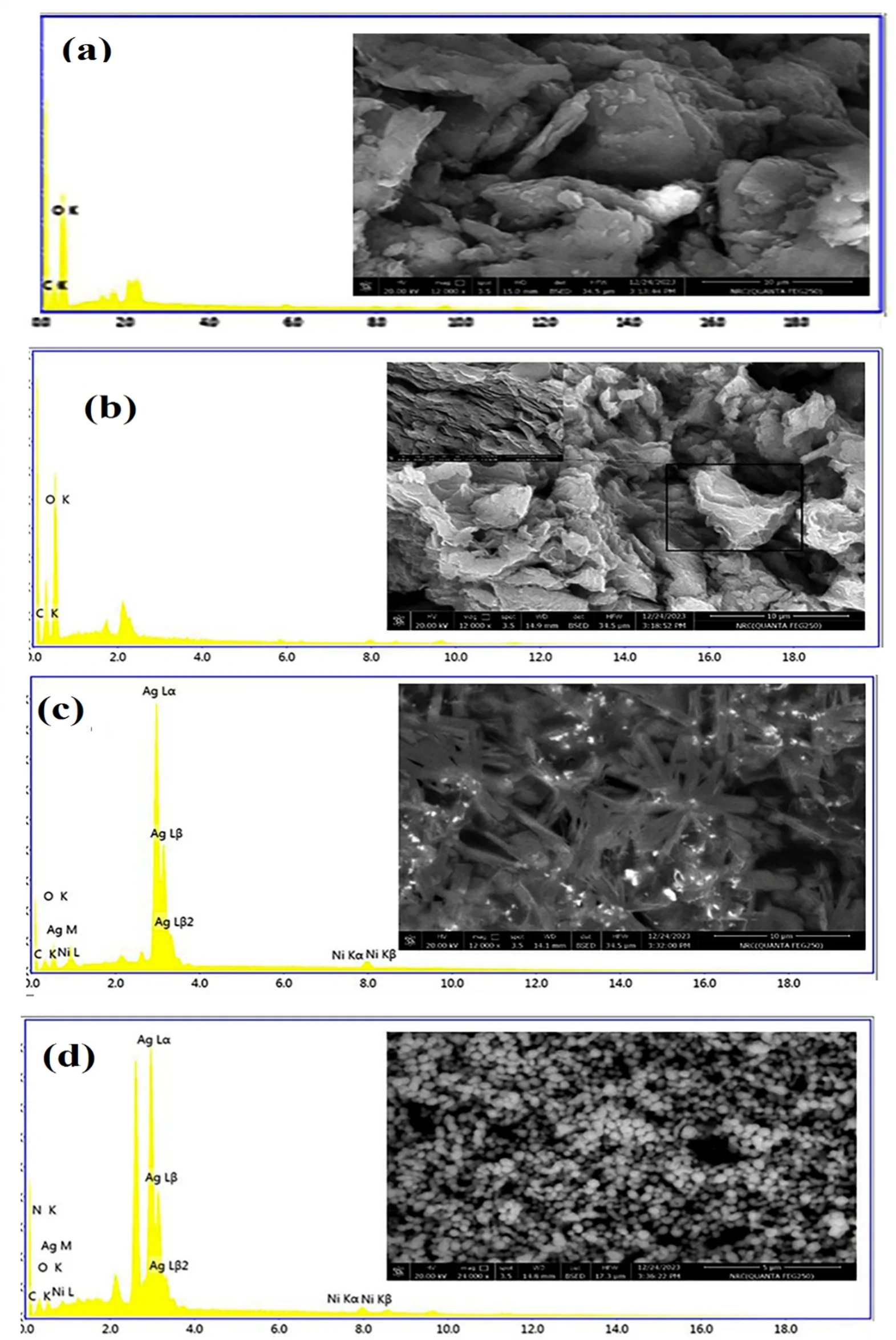
SEM/EDAX of (**a**) oCB, (**b**) roCB, (**c**) Ag_2_O/NiO/roCB, and (**d**) Ag_2_O/NiO/roCB@ PANi.

**Fig. 6:**
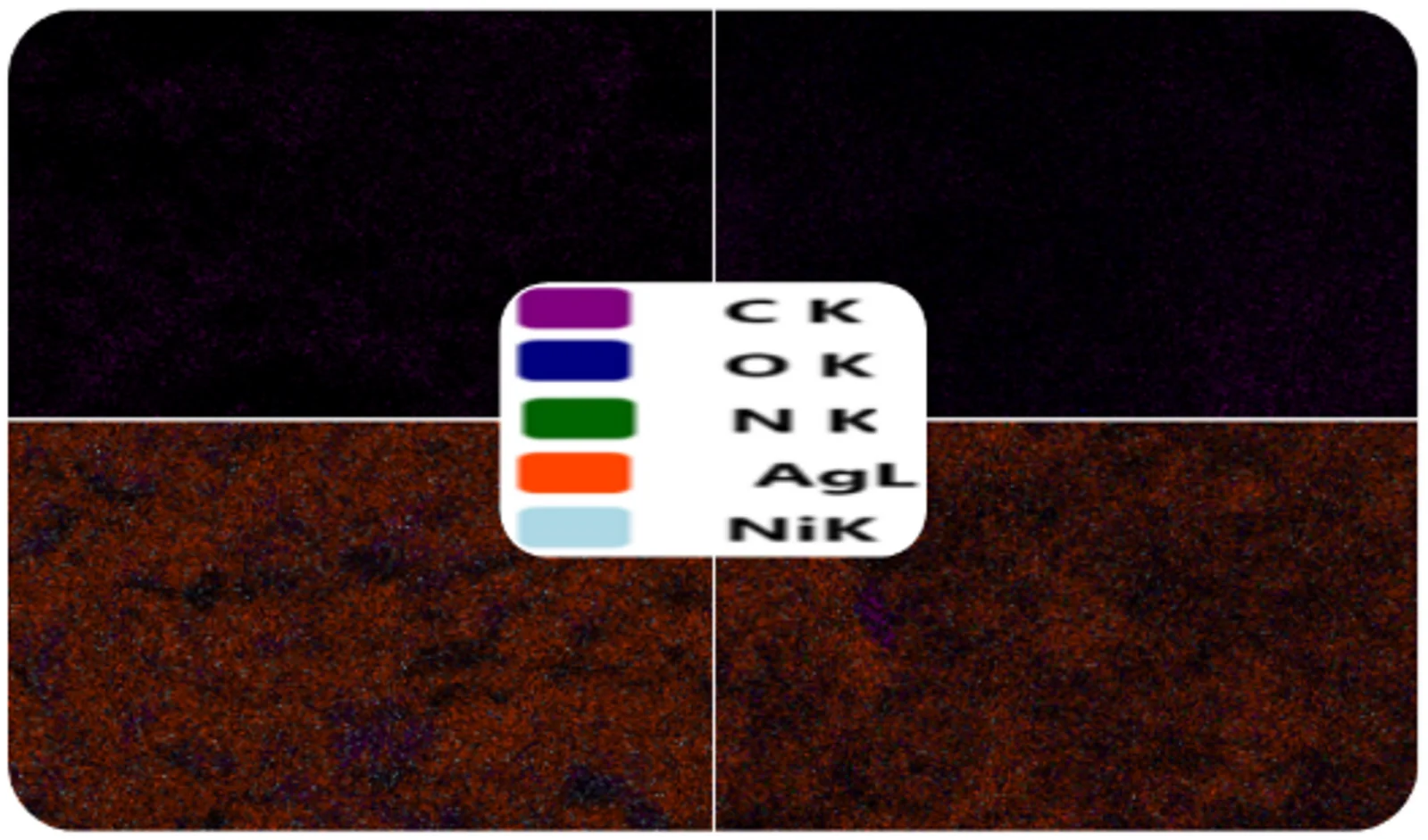
Elemental mapping images of (**a**) oCB, (**b**) roCB, (**c**) Ag_2_O/NiO/roCB, and (**d**) Ag_2_O/NiO/roCB@ PANi.

Figures 5 and 6 depict SEM/EDAX and elemental mapping images of oCB, roCB, Ag_2_O/NiO/roCB, and Ag_2_O/NiO/roCB@ PANi. Figure 5a and b show clustered sheet formations of oCB, with EDAX analysis revealing carbon and oxygen atomic percentages of 99.97% and 0.03%, respectively, confirming the oxidation of CB. Figure 5c and d display a layered crystalline structure, indicating exfoliation in the roCB sample, consistent with XRD findings. The reduction of oxygen content to 0.02% confirms the successful reduction of oCB. Figure 4e and f present Ag_2_O/NiO/roCB as flower-like structures, with EDAX showing atomic percentages of C (84.78%), O (12.19%), Ag (0.03%), and Ni (3%). Figure 4g and h depict Ag_2_O/NiO/roCB@ PANi forming spherical clusters, with proportional elemental compositions of C (78.95%), O (5.06%), N (13.61%), Ag (0.03%), and Ni (2.35%).

Figure 6 demonstrates the distribution of elements within the samples, highlighting oxygen distribution in oCB and roCB (Fig. 6a and b), confirming oxidation and reduction processes. Figure 6c illustrates the distribution of C, O, Ag, and Ni in Ag_2_O/NiO/roCB, while Fig. 6d presents C, O, N, Ag, and Ni dispersion within the Ag_2_O/NiO/roCB@ PANi matrix.

**Fig. 7:**
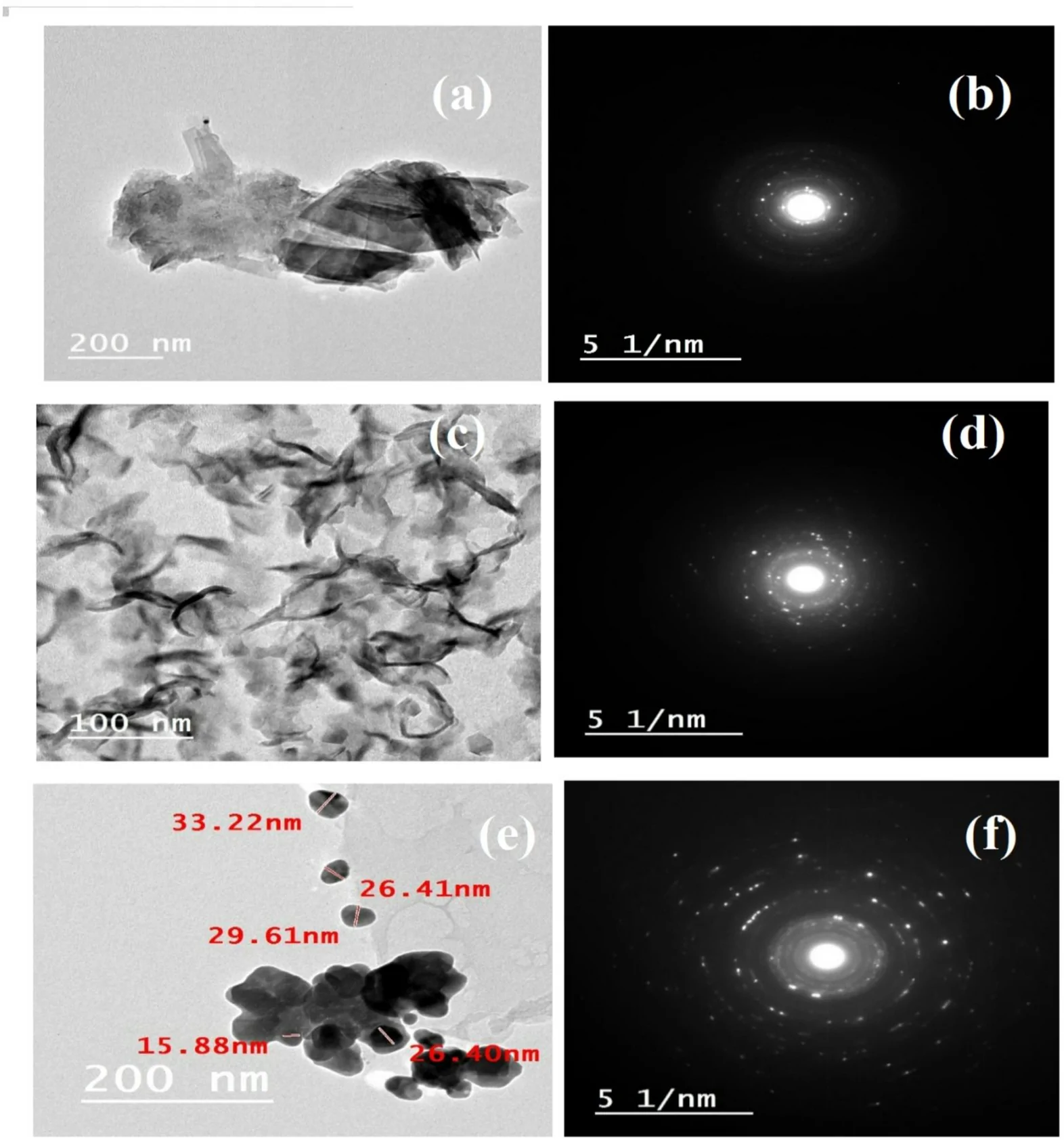
TEM and selected electron diffraction area of roCB (**a**, and **b**), Ag_2_O/NiO/roCB (**c**, and **d**), and Ag_2_O/NiO/roCB@ PANi (**e**, and **f**).

Figure 7 shows TEM images of roCB, Ag_2_O/NiO/roCB, and Ag_2_O/NiO/roCB@ PANi. Figure 6a and c, and 6e reveal sheet-like, rod-shaped, and spherical structures, with particle sizes measuring 26.3 nm for roCB, Ag_2_O/NiO/roCB, and Ag_2_O/NiO/roCB@ PANi, respectively. Figure 6b and d, and 6f illustrate selected electron diffraction areas, confirming nanocrystalline structures consistent with XRD analyses.

### Fluorescence microscope

**Fig. 8:**
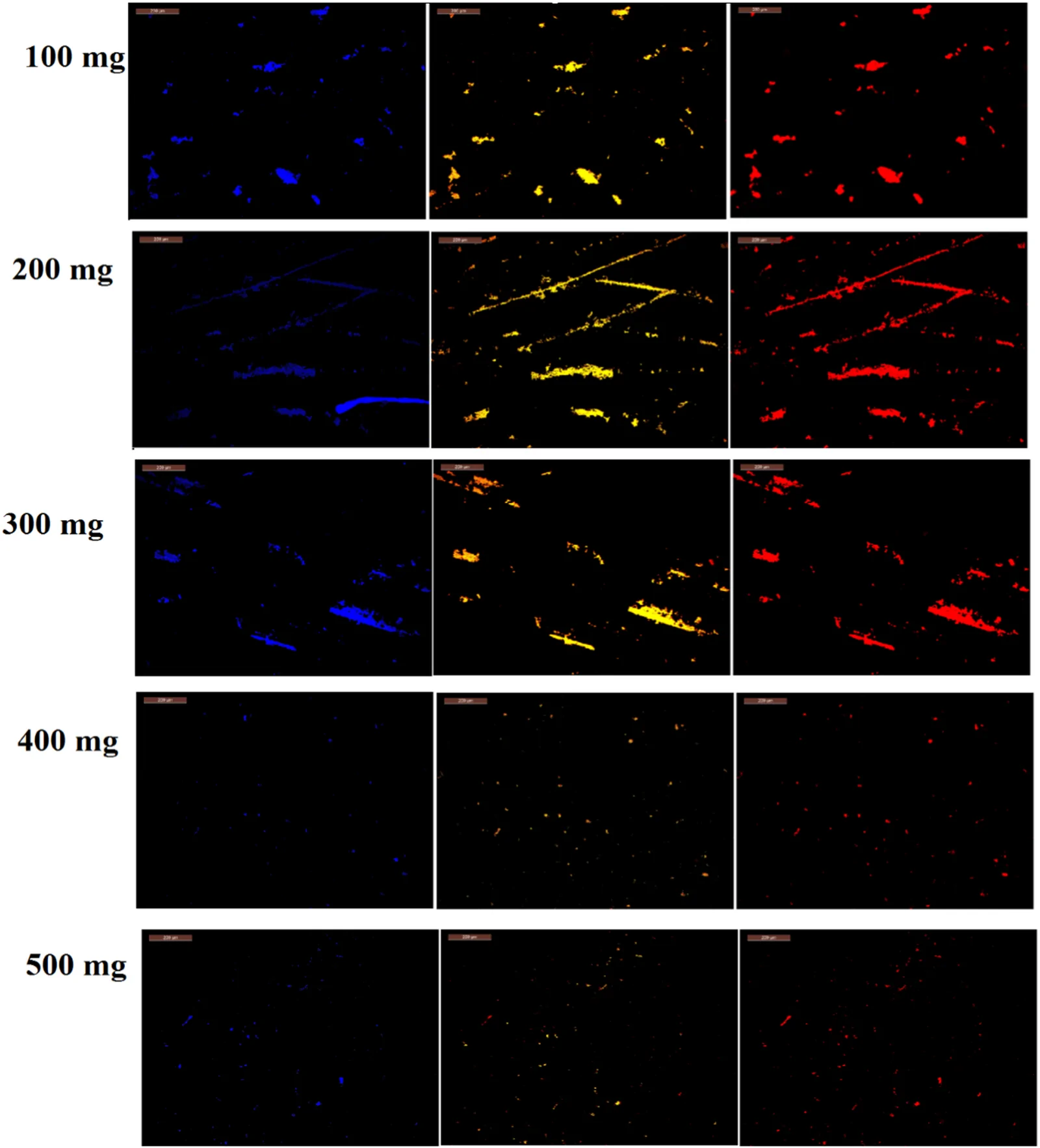
Flouresence microscope images of the Ag_2_O/NiO/roCB@ PANi nanocomposites of different concentrations (100, 200, 300, 400, 500 mg).

Fluorescence is the process through which a substance emits light after absorbing it. The wavelength of the emitted light is often longer than the wavelength of the absorbed light. The current fluorophores, which are in charge of light absorption, absorb light of a specific wavelength and emit light of longer wavelengths when a substance is irradiated by light (i.e., of a dissimilar color from the absorbed one). The optical response of the produced composites to light radiation was examined by the use of a fluorescence microscope examination. The prepared composites are exposed to incident light of different wavelengths from a fluorescence microscope in order to determine the associated changes in their electronic/optical states resulting from charge separation and transport as a result of the incident light on them. Figure 8 shows the examination and illustration of the fluorescence characteristics of the created nanocomposites using a fluorescence microscope under irradiation by three different wave lengths (blue, green, and red). Figure 8 showed that the nanocomposites emit orange red light when exposed to red light, yellowish green when exposed to green light, and violet blue when exposed to blue light. This suggests that the produced nanocomposites contain fluorophores that are both light-absorbing and capable of longer-wavelength light reemission. These characteristics support their application in LEDs, solar cells, and electronic devices.

### Cyclic voltammetric measurements

Polyaniline (PANI), a widely studied conducting polymer, has found broad applications in electrochemical energy storage and conversion systems- such as supercapacitors, rechargeable batteries, and fuel cells- due to its high electrical conductivity, ease of synthesis, mechanical flexibility, cost- effectiveness, and distinctive redox properties. However, its long-term operational stability remains a key limitation^20^.

Recent studies have increasing focused on improving electrochemical performance of polyaniline (PANI) by developing composite electrodes and supported electrocatalysts modified for fuel cells applications. In particular, the combination of conducting polymers with inorganic nanoparticles has attracted considerable interest, as such hybrids exhibit enhanced physical and electrochemical characteristics. Incorporating inorganic fillers or carbon based materials into the PANI matrix offers an effective strategy to optimize its conductivity and broaden its functional capabilities^21^.

These limitations can be effectively mitigated by incorporation of carbon-based materials and metal nanoparticles into the PANI matrix effectively improved its electrochemical behavior and stability. The resulting PANI-based composites exhibited enhanced charge transport and redox activity, attributed to synergistic interactions between the polymer and the conductive fillers. These improvements were particularly evident in fuel cell-related tests, where the composites outperformed pristine PANI in both conductivity and catalytic response^32^.

Cyclic voltammetry (CV) is a widely used technique for evaluating the redox behavior of organic materials by identifying their oxidation and reduction potentials. These values are instrumental in estimating the energy level of the highest occupied molecular orbital HOMO represents the energy required to remove an electron from a molecule (oxidation ) and the lowest unoccupied molecular orbital LUMO energy levels corresponds to the energy needed to add an electron to a molecule (reduction)^24,33^, which correlated with the onset of the oxidation and reduction peaks in the voltammogram. The onsets potentials are commonly determined by the intersection of the tangent drawn at the rising edge of the redox current with the baseline current. This approach also allows for estimation of the energy band gap^34^.

**Fig. 9:**
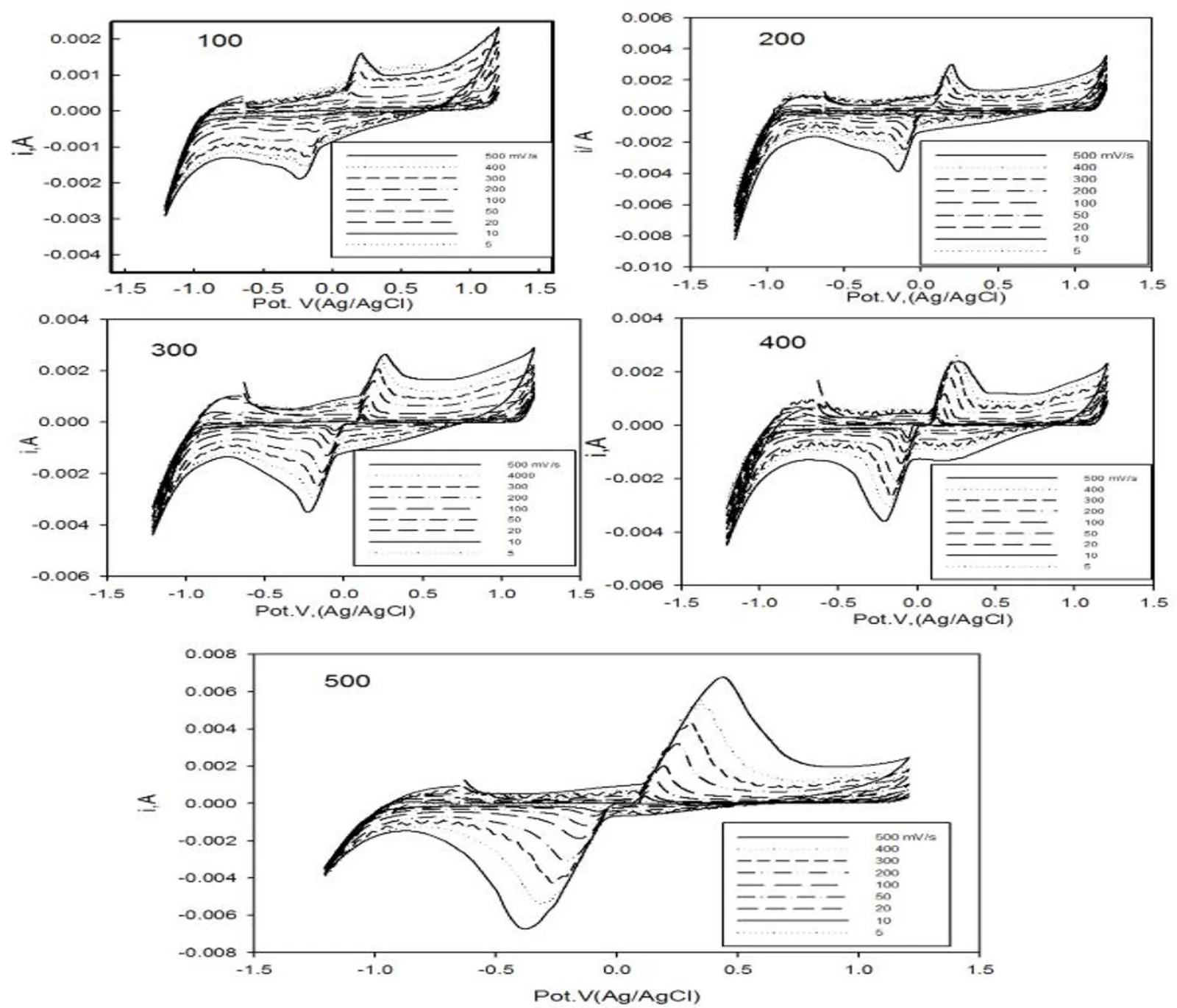
Cyclic voltammograms of different concentration of Ag_2_O/NiO/roCB@ PANi nanocomposite with different scan rate 500 to 5 mVs^− 1^.

Figure 9 displays the cyclic voltammograms for all samples recorded in a 0.1 M KCl solution at scan rates ranging 5 mVs^− 1^ to 500 mVs^− 1^. The CV curves illustrate the relationship between the applied potential (x-axis) and the measured current response (y-axis). The potential is initially swept in the positive direction to capture the oxidation behavior, and then reversed to monitor the corresponding reduction response.

The electrochemical performance of the Ag_2_O/NiO/roCB@ PANi composite was investigated through cyclic voltammetry at Ag_2_O/NiO/roCB concentrations ranging from 100 to 500 mg, offering valuable insights into its redox behavior, stability, and potential electrochemical applications. Analyzing the redox peaks is essential for evaluating and enhancing the material’s performance^35^.

The cyclic voltammograms recorded at different concentrations (100 to 500 mg) show a pronounced dependence of the electrochemical response on the amount of Ag_2_O/NiO/roCB present in the electrolyte. As the scan rate increases, both anodic and cathodic peak current increase. CV behavior confirms that the Ag_2_O/NiO/roCB@ PANi composite exhibits good electrochemical activity over a wide range of scan rate, which is essential for its application in electrocatalytic processes.

As shown from Fig. 9, the CV curves of S 500 exhibit significantly higher anodic and cathodic currents and more distinct redox features compared to those obtained from sample S100, indicating a larger population of electrochemically active species which in turn enhance the participating in the charge-transfer process. This behavior indicates that higher concentration of (Ag_2_O/NiO/roCB) provides a larger number of electroactive species which enhances the active sites and the interfacial charge transfer, which in turn recommend their use in fuel cells and solar cell.

In the Ag_2_O/NiO/roCB@ PANi composite, the anodic sweep of the cyclic voltammogram reveals the oxidation of polyaniline to its higher oxidation states, possibly accompanied by the oxidation of nickel and silver species. During reverse scan, corresponding cathodic peaks appear indicating the reduction of these oxidized forms and confirming the redox reversibility of the composite system.

The position of each peak provides insight into the electron transfer, lower onset potentials reflect greater ease of oxidation or reduction requiring less energy input. Additionally, the peak current intensity correlates with charge density and active species concentration, with higher peaks suggesting favorable reactions or increased reactant concentrations.

The cathodic peak corresponds to reduction of oxidized polyaniline back to its original, reduced form, and may also reflect the reduction of nickel and silver ions to their metallic states. This step offers important insights into the redox reversibility and stability of the composite system, highlighting how ready the metal ions can return to their neutral form.

As shown in Fig. 8, the reproducibility of redox of peak positions and current intensities over successive scans confirms the electrochemical stability of the Ag_2_O/NiO/roCB@ PANi composite, an essential factor for assessing its suitability in long-term fuel cell and electrocatalytic applications.

**Fig. 10:**
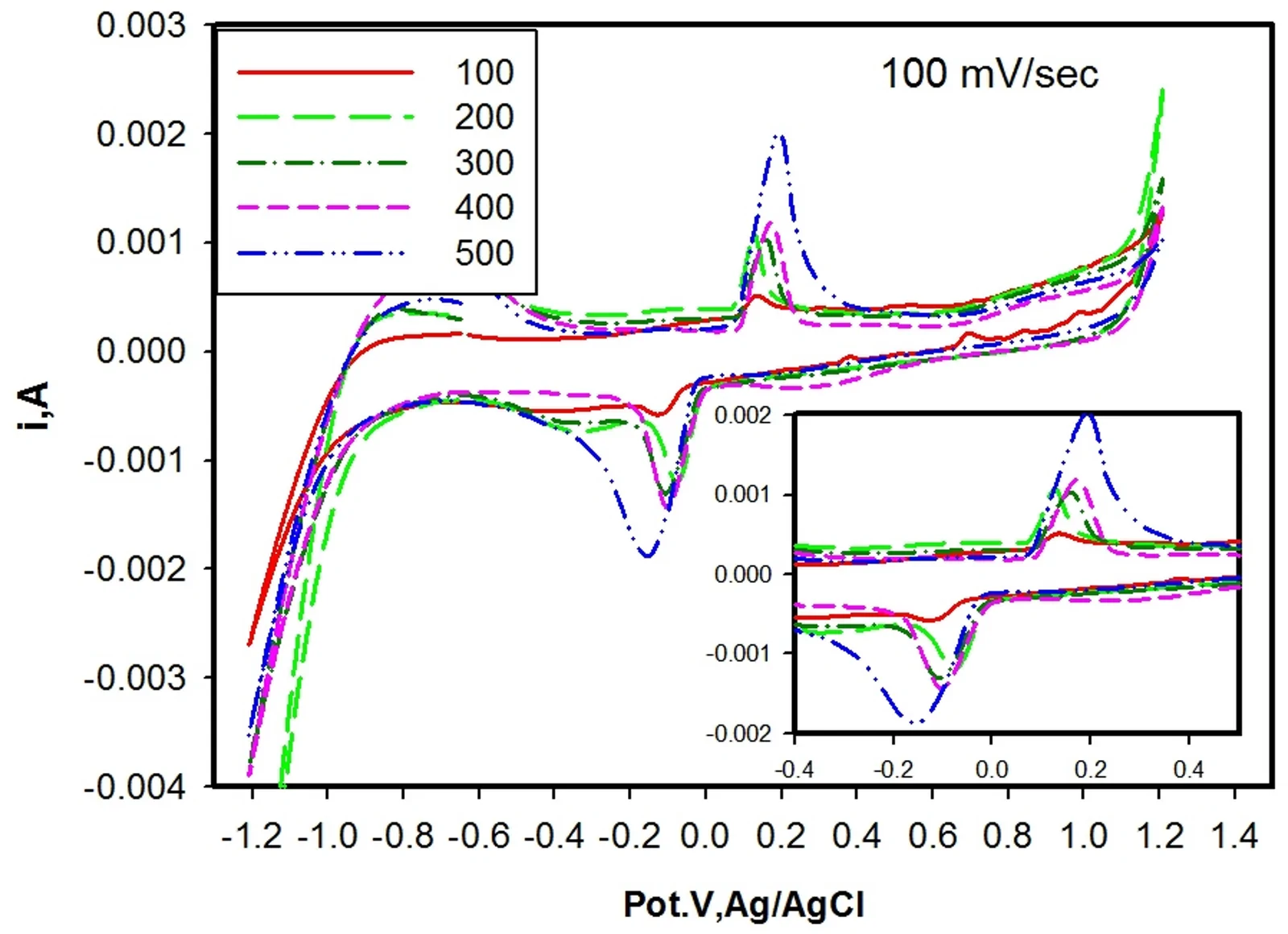
Cyclic voltammograms of different concentration of Ag/Ni/roCB@ PANi nanocomposite with scan rate 100 mV.s^− 1^.

As seen in Fig. 10, using the onset oxidation and reduction potentials (+ 0.090 V and − 0.020 V vs. Ag/AgCl), the HOMO and LUMO energy levels were determined to be − 4.490 eV and − 4.380 eV, respectively, relative to the vacuum level. The resulting electrochemical band gap of 0.11 eV arises from the presence of metallic species and conductive carbon network, which introduce intermediate electronic states, making it well-suited for rapid electron transport in electrocatalytic processes such as hydrogen evolution and fuel cell applications^18,22^.

The HOMO and LUMO levels estimated from cyclic voltammetry mainly reflect the redox states of PANI and metal-polymer interfaces rather than the true band structure of the composite^23^.

Overall, the Ag_2_O/NiO/roCB@ PANi composite demonstrated enhanced electrochemical behavior. This improved performance is primarily due to the excellent conductivity of the carbon-based component, which increased the number of active sites and facilitated more efficient electron transfer. These results highlight the promise of polyaniline-based nanocomposites especially those integrating metal nanoparticles and conductive carbon for advancing hydrogen evolution technologies and fuel cell applications.

Electrochemical impedance spectroscopy (EIS) was used to investigate the interfacial charge transfer of Ag_2_O/NiO/roCB@ PANi composite film, at open circuit potential OCP over a frequency range 100 kHz to 0.01 Hz using sinusoidal AC perturbation of 5 mV, Nova 1.11 software was used to analyze the data.

**Fig. 11:**
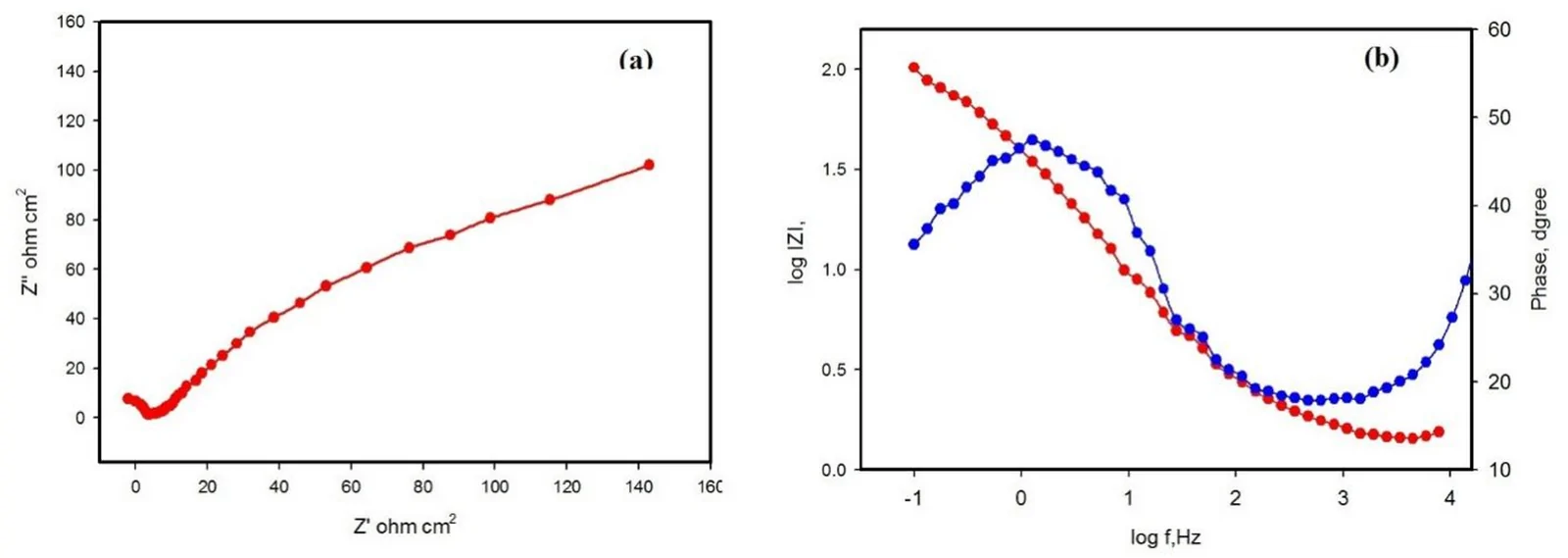
EIS plots for Ag_2_O/NiO/roCB@ PANi in 1 M KOH (**a**) Nyquist plots, (**b**) bode and phase plots.

Figure 11 shows the Nyquist and Bode plots of the Ag_2_O/NiO/roCB@ PANi composite. The Nyquist plot shows low semicircle in the high frequency region, the relatively small diameter of the semicircle indicates a low charge transfer resistance (R_ct_), representing efficient electron transfer across the electrode/electrolyte interface during hydrogen evolution reaction. The low semicircle suggests non-ideal capacitive behavior resulting from the heterogeneous surface^36^.

The Bode plots further approve electrochemical behavior of the composite. The impedance modules IZI decreases with increased frequency indicating low impedance and efficient charge transfer through the electrode. The phase angle-plot exhibits a broad peak at intermediate frequencies; indicate that the hydrogen evolution process is governed by a single charge transfer time constant.

The enhanced electrochemical performance can be attributed to the synergistic addition of Ag_2_O, NiO, reduced carbon black, and the conducting PANI matrix. The roCB provides highly conductive pathways that facilitate rapid electron transport, while the Ag_2_O further reduces electronic resistance due to excellent electrical conductivity. The incorporation of NiO introduced additionally catalytically active sites that promote hydrogen adsorption and proton reduction. PANI matrix serves as an efficient conductive matrix that increases the electronic coupling between metallic nanoparticles and the carbon support. These collective effects decrease the interfacial charge transfer resistance, accelerate electron-transfer and facilitating the hydrogen evolution reaction^37^.

The EIS analysis demonstrates that the composite possesses low interfacial charge transfer resistance, highly electrical conductivity, and efficient charge transport leading to promoting rapid electron transfer to the active site and hence enhancing the hydrogen evolution electrocatalytic performance.

### Dielectric study

The dielectric constant (έ) and AC electrical conductivity (σac) of Ag_2_O/NiO/roCB@ PANi were examined at Ag_2_O/NiO/roCB concentrations of 100, 200, 300, 400, and 500 mg across a frequency range of 42 Hz to 1 MHz at room temperature.

The dielectric spectroscopic study provides valuable insights into the structural composition, grain boundary characteristics, transport mechanisms, and charge storage capabilities of dielectric materials. These properties are influenced by several factors, including the chemical composition and preparation method^38,39^.

**Fig. 12:**
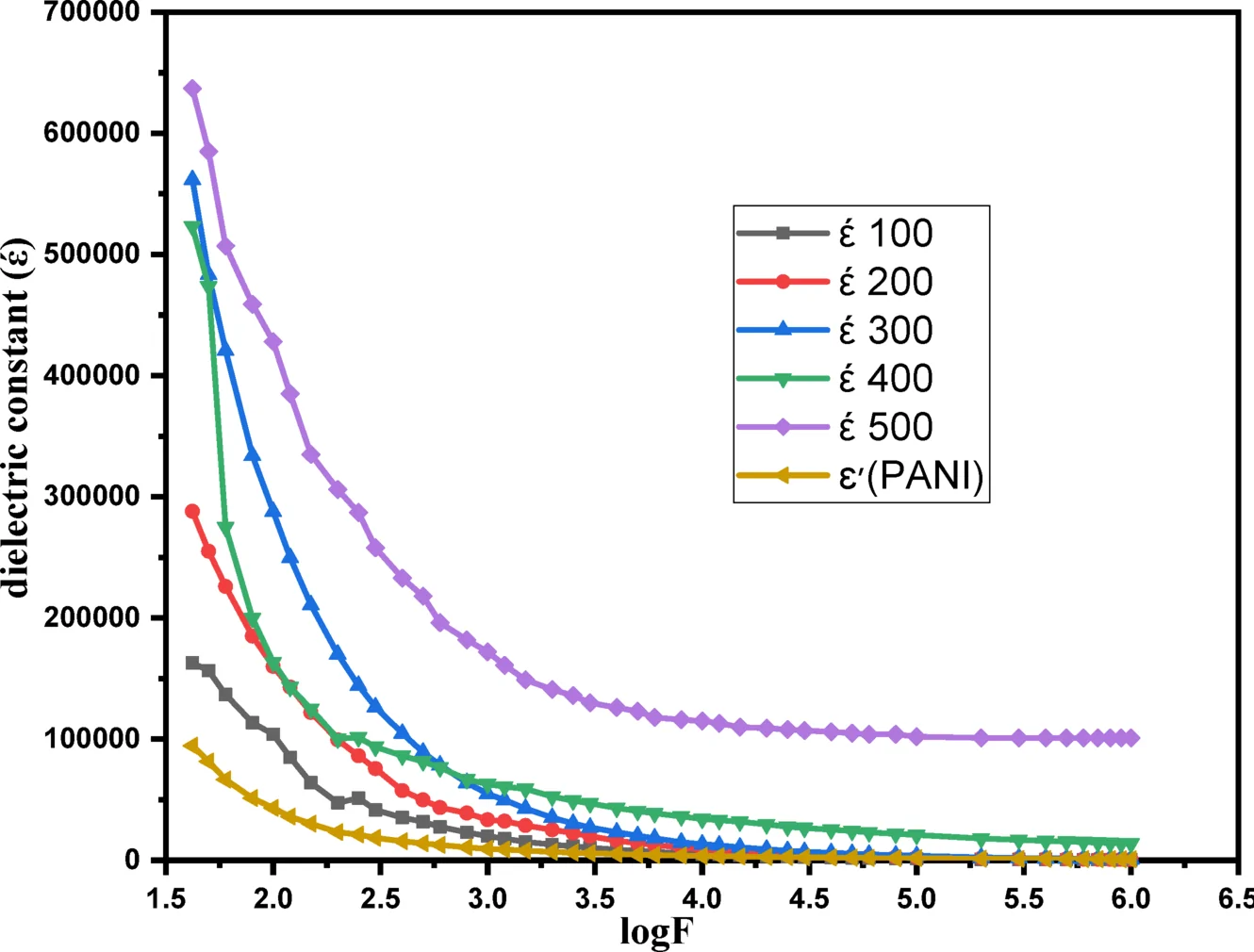
The variation of dielectric constant of Ag_2_O/NiO/roCB@ PANi nanocomposites with different ratio of doping with frequency at room temperature.

Figure 12 illustrates the frequency-dependent behavior of the dielectric constant (έ) for the studied composites within a broad frequency range (42 Hz to 1 MHz). In this study, the real part of the dielectric constant (ε′) was determined from capacitance measurements at room temperature, using the following equation:

1 $$ \varepsilon \prime {\text{ }} = {\text{ C}}_{{\mathrm{p}}} {\text{d }}/\varepsilon _{{\mathrm{o}}} {\mathrm{A}}~ $$

where ε_0_ represents the permittivity of free space, d is the thickness of the pressed disc, and A denotes the surface area under examination. The results indicate that the dielectric constant decreases as frequency increases. At lower frequencies, this elevated dielectric constant is attributed to the contributions of various polarization mechanisms, including space charge, dipolar, ionic, and electronic effects^40,41^. Moreover, a sharp decline in ε′ within the low-frequency region is primarily due to intensified interfacial space polarization^42^. The observed dielectric dispersion can be explained by the dominant effect of grain boundaries rather than individual grains. This behavior aligns with the Maxwell-Wagner interfacial polarization model and Koop’s phenomenological theory^43^, which postulate that dielectric structures comprise low-resistance grains separated by poorly conductive thin grain boundaries. Under an applied electric field, charge accumulation occurs, leading to interfacial polarization^44,45^.

Overall, the dielectric constant values are significantly high due to the presence of polyaniline (PANi), a conducting polymer with semiconducting properties. Additionally, the incorporation of Ag and Ni ions, along with CB, enhances the dielectric constant further. The incorporation of roCB into metal oxide and polymer–based composites eliminates electron – hole (e-/h*) recombination and narrows energy band gaps, significantly increasing the electrical conductivity properties of the prepared composites. The presence of roCB together with nanometal oxides promotes strong interfacial bonding with semiconductor nanoparticles of PANI, enhancing charge separation and transport^46–48^. Specifically, the dielectric constant of pure PANI at 42 Hz is 6.14 × 10^4^, while that of roCB is 7.08 × 10^2^. As depicted in Fig. 12, the variation in dielectric constant with Ag_2_O/NiO/roCB concentration shows a steady increase. The addition of 100 wt% dopant into PANi raises the dielectric constant from 6.14 × 10^4^ to 1.63 × 10^5^, and as the dopant concentration continues to rise, the dielectric constant at room temperature progressively increases to 2.88 × 10^5^, 5.62 × 10^5^, 5.23 × 10^5^, 6.37 × 10^5^ for S200, S300, S400, and S500 samples, respectively. This trend reflects enhanced interfacial polarization and local electric field amplification, arising from the high aspect ratio and conductive nature of the dispersed species of Ag_2_O, NiO, and roCB^49^.

These findings suggest that the observed enhancement in dielectric constant may be a result of increased conductivity. The improved dielectric properties of Ag_2_O/NiO/roCB@ PANi nanocomposites indicate their superior ability to store electrical potential energy under alternating electric fields. Furthermore, the strong dependence of dielectric constant on frequency, particularly at lower frequencies, underscores the role of multiple polarization mechanisms, including space charge, dipolar, ionic, and electronic effects. The substantial dielectric enhancement resulting from Ag_2_O/NiO/roCB doping supports improved charge storage and interfacial polarization, key factors in optimizing electrolyte interactions for hydrogen evolution applications. The synergistic effect of polyaniline and metallic dopants strengthens charge redistribution within the composite, potentially enhancing the kinetics of hydrogen evolution reactions (HER)^50,51^.

### Ac- electrical conductivity study

The dependence of AC conductivity on frequency was analyzed for the prepared samples. The relationship between conductivity and frequency follows the power law, which is expressed as^52^:

2 $$ \sigma _{{ac}} \left( \omega \right){\text{ }} = {\text{ }}\sigma _{{dc}} + {\text{ }}A\omega ^{s} $$

where σ_dc_ represents the DC conductivity (which remains constant in the low-frequency region), and s is the fractional exponent that reflects the degree of interaction between mobile ions and their surrounding environment^53,54^. In general, AC electrical conductivity in semiconductors follows the universal Jonscher power law^55^.

3 $$ \sigma _{{{\mathrm{ac}}}} = {\text{ A}}\omega ^{{\mathrm{s}}} $$

where A is a temperature-dependent constant, ω (omega) is the angular frequency, and s is the frequency exponent. The values of s are derived from the slopes of the conductivity σ_ac_ versus frequency plots. Table (1) records these values, ranging from 0.0864 for S100 to 0.0452 for S500. It is noteworthy that all recorded s values are below unity, suggesting that the conduction mechanism follows the correlated barrier hopping (CBH) model^56^. According to this model, charge transport occurs through a bipolaron hopping process, wherein two polarons simultaneously hop over the potential barrier formed between oppositely charged defect states D + and D−. The barrier height is influenced by the Coulombic interaction and the separation between defect sites.

Furthermore, dielectric studies reveal a strong dependence of the dielectric constant (ε′) on frequency. At lower frequencies, high dielectric constant values are observed due to contributions from various polarization mechanisms, including space charge, dipolar, ionic, and electronic effects. The substantial increase in dielectric constant upon doping with Ag_2_O/NiO/roCB indicates improved charge storage and enhanced interfacial polarization which are the key factors for optimizing electrolyte interactions in hydrogen evolution systems. The presence of polyaniline, a conducting polymer, in conjunction with metal ions significantly enhances charge redistribution within the composite, potentially accelerating hydrogen evolution reaction (HER) kinetics. The enhanced conductivity achieved by metallic phases of dopants lowers the overpotential required for hydrogen production leading to improving electrocatalytic efficiency. High conductivity electrodes combined with large surface areas are the key to developing low-cost, efficient hydrogen energy reaction HER catalysts.

**Fig. 13:**
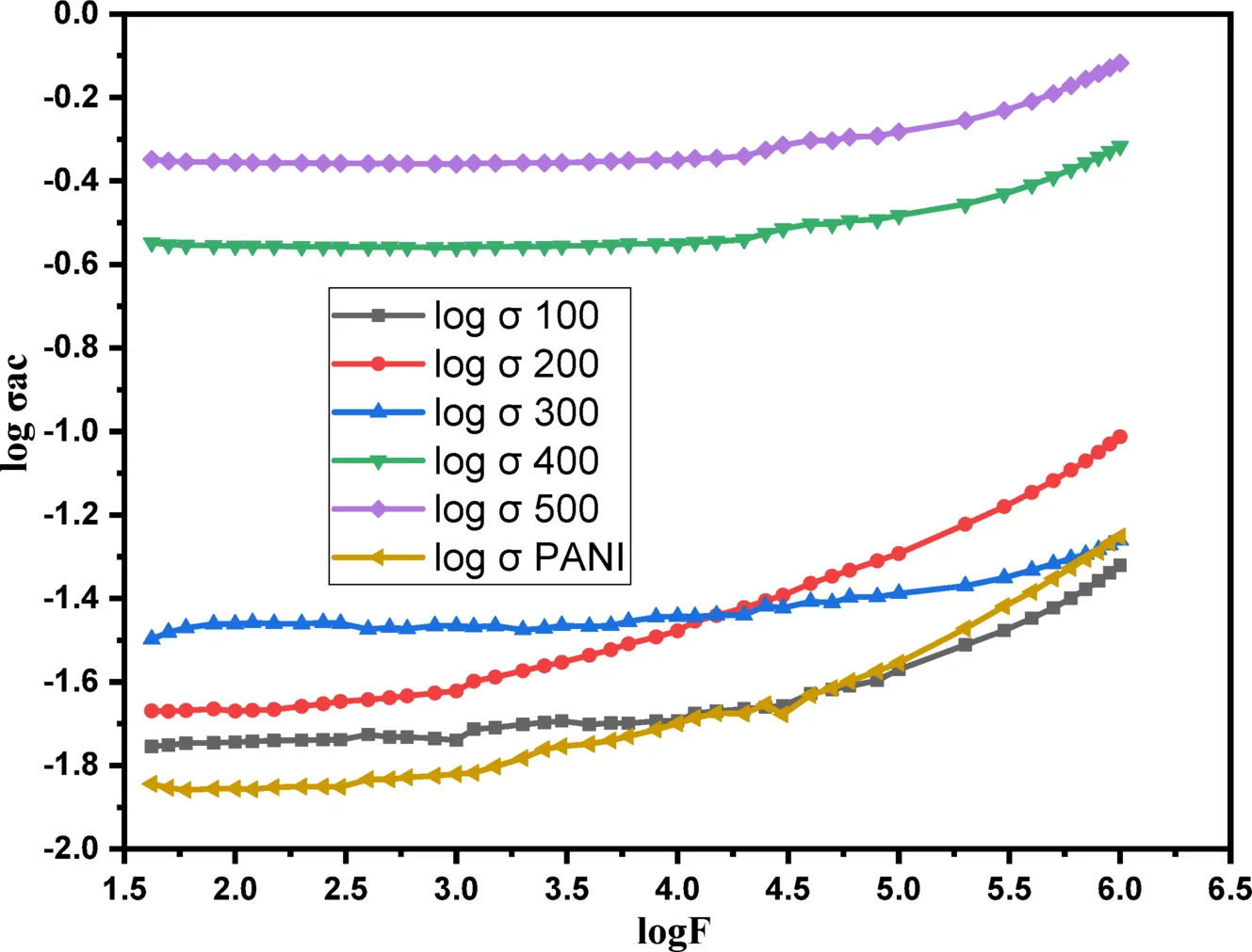
The variation of of Ac-electrical conductivity with frequency at room temperature for Ag_2_O/NiO/roCB@ PANi nanocomposites.

The semiconductor behavior of the synthesized nanocomposites was examined at room temperature across a frequency range of 42 Hz to 1 MHz. The graphical representation in Fig. 13 demonstrates that the AC electrical conductivity of Ag_2_O/NiO/roCB@ PANi nanocomposites is significantly higher than that of pure PANI and roCB. The lower conductivity observed in pure PANI can be attributed to its low protonation level, which limits charge transport. In contrast, the improved AC conductivity of the Ag_2_O/NiO/roCB@ PANi composites is primarily due to the homogeneous dispersion of Ag_2_O/NiO/roCB nanoparticles within the PANI matrix, facilitating enhanced electronic transport.

It is well established that AC conductivity is frequency-dependent and increases with rising frequency. This trend suggests the presence of charge carriers capable of hopping through defect sites along polymer chains^57–59^. Charge transport within these nanocomposites is governed by the ability of charge carriers to move from one molecule to another. Due to the quantum mechanical hopping nature of this transport and its reliance on probability functions, it is often referred to as hopping transport^60,61^.

As observed, the conductivity of nanocomposites increases with frequency. This frequency-dependent behavior is attributed to interface charge polarization (Maxwell-Wagner-Sillars effect) and intrinsic dipole polarization. Such phenomena are commonly observed in heterogeneous systems such as metal-polymer composites, where mobile charge accumulates at interfaces, leading to dipole formation on metal particles or clusters. Various theoretical models describe AC conductivity in amorphous semiconductors depending on the value of s, including classical hopping and quantum mechanical tunneling, where charge carriers overcome potential barriers separating energetically favorable sites in a random distribution^62^.

**Table 1:** The values of dielectric constant ε′, AC-electrical conductivity of different frequency range in addition to the values of frequency exponents (S) for the prepared Ag_2_O/NiO/roCB@ PANi nanocomposite.

Samples	s	ε′			σ( scm^− 1^)		
		42 Hz	1 KHz	1 MHz	42 Hz	1 KHz	1 Hz
PANI	0.1345	9.46*10^4^	3.55*10^3^	8.44	1.43*10^− 2^	1.51*10^− 2^	2.93*10^− 2^
roCB	0.3551	501.0	398.3	14.337	2.53*10^− 7^	2.31*10^− 6^	1.05*10^− 5^
S100	0.0864	1.629*10^5^	5.04*10^3^	371.04	1.6*10^− 2^	2.82*10^− 2^	2.689*10^− 2^
S200	0.1515	2.88*10^5^	9.3*10^3^	7.99*10^2^	2.14*10^− 2^	2.38*10^− 2^	5.09*10^− 2^
S300	0.0421	5.617*10^5^	1.33*10^4^	1.01*10^3^	3.18*10^− 2^	3.42*10^− 2^	4.09*10^− 2^
S400	0.0452	5.23*10^5^	3.40*10^4^	1.38*10^4^	2.83*10^− 1^	2.75*10^− 1^	3.29*10^− 1^
S500	0.0452	6.37*10^5^	1.15*10^5^	1.01*10^3^	4.88*10^− 1^	4.36*10^− 1^	5.21*10^− 1^

## Conclusion

This study successfully synthesized and characterized Ag2O/NiO/roCB@ PANi nanocomposites, demonstrating their potential as efficient electrocatalysts for hydrogen evolution reaction (HER). The integration of polyaniline, carbon black, and metal nanoparticles resulted in a synergistic enhancement of electrochemical performance, conductivity, and charge transfer properties. Structural and morphological analyses confirmed the successful formation of conductive nanocomposites, while cyclic voltammetry (CV) revealed distinct redox peaks, providing insight into their stability and catalytic activity. As the concentration of the active species of Ag_2_O/NiO/roCB@ PANi increases from 100 to 500 wt%, the cyclic voltammograms show a clear rise in anodic and cathodic peak currents and more pronounced redox features, indicating an increased number of electroactive species. Effective electron transport at the electrode/electrolyte interface is shown by low charge transfer resistance (Rct) in electrochemical impedance spectra (EIS), which is suitable for the hydrogen evolution process (HER). The combined CV and EIS data show a stronger correlation between the composite’s structure and its electrochemical performance.

This enhanced electrochemical response reflects a higher density of accessible catalytic sites and improved charge-transfer capability, both of which are critical for the hydrogen evolution reaction (HER). Dielectric spectroscopy highlighted a strong frequency-dependent variation in the dielectric constant (ε′), indicating improved charge storage and interfacial polarization due to Ag_2_O/NiO/roCB@ PANi doping. AC conductivity (σac) followed the correlated barrier hopping (CBH) model, confirming enhanced charge transport across the nanocomposite matrix. The FM results revealed that the nanocomposites contain fluorophores that are both light-absorbing and capable of longer-wavelength light. These characteristics support their application in LEDs, solar cells, and electronic devices. The results showed that the nanocomposite S500 is the most appropriate candidate to be used as it has the highest conductivity. Higher electrical conductivity in catalysts directly accelerates the HER by facilitating faster electron transfer from the electrode to reactant species, reducing charge-transfer resistance. Enhanced conductivity of the prepared nanocomposites lowers the overpotential required for hydrogen production and improves the electrocatalytic efficiency. From the obtained results, one can conclude that the Ag_2_O/NiO/roCB@ PANi nanocomposites provide an affordable and efficient electroactive materials.

The novelty of this research lies in the integration of conductive polymers, reduced carbon black, and metallic catalysts to achieve promising electrochemical as well as optical properties which will contribute to advancements in sustainable hydrogen production as well as photovoltaics technologies, offering new possibilities for clean energy applications.

## Data Availability

All data generated or analyzed during this study are included in the current manuscript.
